# Novel Anti-Enterovirus A71 Compounds Discovered by Repositioning Antivirals from the Open-Source MMV Pandemic Response Box

**DOI:** 10.3390/ph17060785

**Published:** 2024-06-14

**Authors:** Nattinee Lochaiyakun, Potjanee Srimanote, Onruedee Khantisitthiporn, Jeeraphong Thanongsaksrikul

**Affiliations:** 1 Graduate Program in Biomedical Sciences, Faculty of Allied Health Sciences, Thammasat University, Pathumthani 12120, Thailand; nattinee.loc@allied.tu.ac.th (N.L.); psrimanote01@yahoo.com.au (P.S.); 2 Thammasat University Research Unit in Molecular Pathogenesis and Immunology of Infectious Diseases, Thammasat University, Pathumthani 12120, Thailand; yuleeko@hotmail.com; 3 Department of Medical Technology, Faculty of Allied Health Sciences, Thammasat University, Pathumthani 12120, Thailand

**Keywords:** enterovirus, enterovirus A71, hand-foot-mouth disease, antivirus drugs, repurposed drugs

## Abstract

The open-source drug library, namely, MMV Pandemic Response Box, contains 153 antiviral agents, a chemically and pharmacologically diverse mixture of early-stage, emerging anti-infective scaffolds, and mature compounds currently undergoing clinical development. Hence, the Pandemic Response Box might contain compounds that bind and interfere with target molecules or cellular pathways that are conserved or shared among the closely related viruses with enterovirus A71 (EV-A71). This study aimed to screen antiviral agents included in the Pandemic Response Box for repurposing to anti-EV-A71 activity and investigate the inhibitory effects of the compounds on viral replication. The compounds’ cytotoxicity and ability to rescue infected cells were determined by % cell survival using an SRB assay. The hit compounds were verified for anti-EV-A71 activity by virus reduction assays for viral RNA copy numbers, viral protein synthesis, and mature particle production using qRT-PCR, Western blot analysis, and CCID_50_ assay, respectively. It was found that some of the hit compounds could reduce EV-A71 genome replication and protein synthesis. D-D7 (2-pyridone-containing human rhinovirus 3C protease inhibitor) exhibited the highest anti-EV-A71 activity. Even though D-D7 has been originally indicated as a polyprotein processing inhibitor of human rhinovirus 3C protease, it could be repurposed as an anti-EV-A71 agent.

## 1. Introduction

Enterovirus A71 (EV-A71) is one of the most common etiological agents of hand-foot-mouth disease (HFMD), besides coxsackievirus A6 (CVA-6), CVA10, and CVA16, that has been frequently associated with neurological complications [1,2,3,4,5]. The HFMD is commonly found in children under the age of 5 years but can affect adults [1]. Clinical manifestations of HFMD include asymptomatic, uncomplicated-, and complicated HFMD [1]. The predominant clinical manifestations of HFMD cases are fever, oral ulceration, vesicular rashes, or small erythematous maculopapular rashes scattered on palms, feet, and buttocks, namely uncomplicated HFMD [1]. EV-A71 infection could progress to develop neurological complications, called complicated HFMD, including meningitis, encephalitis, acute flaccid myelitis (AFM), and neurogenic cardiopulmonary failure [1,2,3,4,5]. It has been estimated that 6% of the symptomatic cases are hospitalized [2].

Among the hospitalized cases, 18.7% of them are expected to develop complications, and 5% of such cases are fatal [2]. EV-A71 has been responsible for the majority of the fatal cases. HFMD outbreaks have been found worldwide, but the high disease burden has been confined to Asia-Pacific countries, which have established endemicity in the area for decades [4,6]. Millions of HFMD cases have been reported annually in Asia, leading to a massive healthcare burden and economic loss [5,6,7]. It has been estimated that the basic reproduction number (R0) for EV-A71, CVA-16, and CVA-6 is 12.60, 15.50, and 25.80, respectively [8]. Hence, the high number of HFMD cases is likely associated with the contagiousness of the causative enteroviruses. With regard to the control of disease spreading and mitigating neurological complications, the need exists to develop protective vaccines, specific treatments, and effective antivirals for EV-A71 infection. Currently, three inactivated whole EV-A71 vaccines have been approved but limited to use only in China [9,10,11]. Unfortunately, antiviral drugs that are effective against EV-A71 have not been found in clinical practice to mitigate neurological complications [12,13].

Even though vaccines are recognized as a preferred promising line of defense against virus outbreaks, vaccine development is a complex process. Multiple challenges are involved in unknown biology and high genetic variability of the pathogen, leading to contemporary unavailability or reducing vaccine efficacy. Therefore, antiviral drugs are more promising for combating those viruses; meanwhile, effective vaccines are in shortage. Nowadays, the discovery of drugs for emerging or re-emerging viruses has been accelerated by exploring new molecular pathways and targets for intervention of the well-characterized drugs, either approved or under investigation, to identify the novel treatment of virus infections that beyond the original indicative scope, namely drug repurposing [14]. Given that the repurposed drugs have been proven to be safe in humans, drug repurposing can reduce not only the attrition rate to be a novel antiviral but also time and resource-consuming for drug development. Drugs with repurposing potential against viral diseases have been identified mostly by screening the small-molecule libraries consisting of approved and investigational drugs, as well as natural compounds [15]. These libraries are available as open-source or commercial. Therefore, it could be expected that a drug repurposing strategy can be employed to discover antiviral drugs against the EV-A71.

In 2019, the Medicines for Malaria Venture (MMV) collaborated with the Drugs for Neglected Diseases *initiative* (DND*i*) to launch an open-source drug library, namely MMV Pandemic Response Box, to accelerate drug discovery efforts against emerging pandemic diseases [16]. The MMV Pandemic Response Box contains 400 compounds, including 201 antibacterial, 46 antifungal, and 153 antiviral agents. These compounds are a chemically and pharmacologically diverse mixture of early-stage, emerging anti-infective scaffolds, and more mature compounds currently undergoing clinical development [16]. A successful repurposing of the compounds in the Pandemic Response Box against Zika and SARS-CoV-2 virus has been reported [17,18]. Nonetheless, there is no report on repurposing the compounds in the Pandemic Response Box to evaluate anti-EV-A71 activity [16]. Importantly, the mode of action for compounds included in the Pandemic Response Box is mostly well-characterized [16]. We hypothesized that the antiviral agents included in the Pandemic Response Box might bind and interfere with target molecules or cellular pathways that are conserved or shared among the closely related viruses with the EV-A71. Hence, this study aimed to screen antiviral agents included in the Pandemic Response Box for repurposing to anti-EV-A71 activity and investigate the inhibitory effects of the compounds on viral replication. The gained knowledge will be beneficial in developing a treatment regimen and opening the therapeutic avenue to counteract EV-A71 infection, a major public health problem in the Asia-Pacific region.

## 2. Results

### 2.1. Evaluation of Cytotoxicity of Antiviral Compounds of the MMV Pandemic Response Box

**Figure 1:**
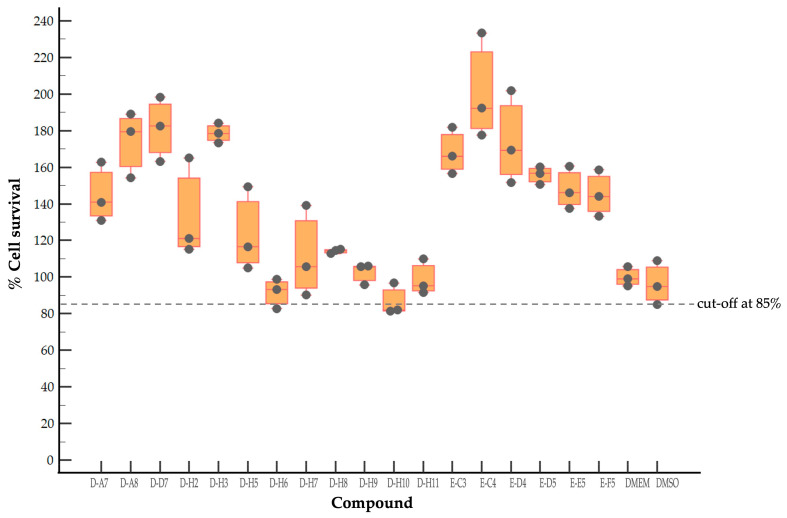
The results of the cytotoxicity test of the selected 18 compounds. RD cell monolayers were incubated with 1 μM of the tested compounds for 24 h. The names of the compounds were according to the location in the plate layout of the Pandemic Response Box (plate-well number). DMEM and DMSO were cell culture medium alone and supplemented with DMSO, served as maximum % cell survival and vehicle control, respectively. Cytotoxicity of the compounds was determined by SRB assay and calculated for % cell survival relative to the maximum % cell survival control. The % cell survival values are derived from three independent experiments and presented in Box-and-whisker and dot plots. Each dark grey circle represents the individual data points, the middle bar is the median, and the boxes represent quartile data distribution. The dashed line represents the cut-off point derived from a mean − 1SD of % cell survival of the DMSO controls.

To evaluate the cytotoxicity of the antiviral compounds, 153 antiviral agents at 1 μM as recommended concentration by the MMV were incubated with rhabdomyosarcoma (RD) cell monolayers, and cytotoxicity was determined by a sulforhodamine B (SRB) assay. RD cells incubated with culture medium alone (DMEM) and supplemented with dimethyl sulfoxide (DMSO) served as maximum % cell survival and vehicle control, respectively. The DMEM controls had 100 ± 4.83% cell survival (Figure 1). The DMSO at the concentration corresponding to the solvent of the tested compounds showed 96.31 ± 10.74% cell survival compared with the maximum % cell survival control (DMEM). The cut-off was derived from the mean − 1 standard deviation (SD) value of % cell survival of the DMSO control group, which is approximately 85%. It was found that 18 of 153 antiviral agents had % cell survival ranging from 86.68 ± 7.76 to 201.07 ± 25.86 (Figure 1). It was suggested that the selected 18 antiviral compounds had negligible cytotoxicity to RD cells and were suitable for further investigations.

### 2.2. Screening of Compounds with Potential Anti-EV-A71 Activity

**Figure 2:**
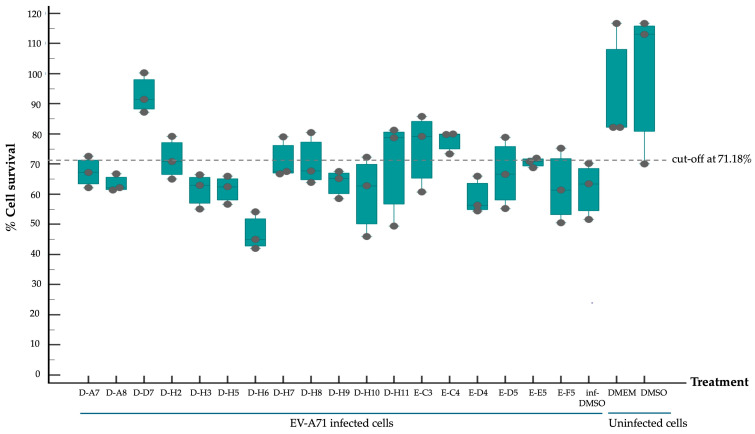
Evaluation of potential anti-EV-A71 activity of the selected 18 antiviral compounds from the MMV Pandemic Response Box. RD cell monolayers were infected with EV-A71 at 1 M.O.I. by adsorbing with the virus inoculum for 1 h and then incubated with fresh medium containing 1 μM of the tested compounds for 24 h. The D-A7 (rupintrivir) and D-H7 (T-1106) compounds served as positive anti-EV-A71 activity control. The infected RD cell monolayers incubated with cell culture medium supplemented with DMSO (infDMSO) served as negative anti-EV-A71 activity control. Uninfected RD cells incubated with cell culture medium alone (DMEM) and DMSO served as maximum % cell survival and vehicle control, respectively. The SRB assay determined % cells rescued from EV-A71-induced cytopathic effect (CPE) and was calculated for % cell survival relative to the maximum % cell survival control. The % cell survival values are derived from three independent experiments and presented in Box-and-whisker and dot plots as described in Figure 1. The dashed line represents the cut-off point relative to the positive anti-EV-A71 activity control (D-H7).

The selected 18 antiviral compounds were determined for anti-EV-A71 activity in RD cells. The RD cell monolayers were inoculated with B5 genotype EV-A71 at 1 multiplicity of infection (M.O.I.). After 1 h, the inoculum was removed, replaced with a fresh culture medium containing 1 μM antiviral compounds as recommended by the MMV, and incubated for 24 h. After a review of the selected compounds’ information, among the selected 18 compounds, two antiviral agents from plate D wells A7 (D-A7) and H7 (D-H7), namely rupintrivir [19] and T-1106 [20], respectively, have been reported for potent anti-EV-A71 activity. Hence, they were used to set cut-off values and were used as a positive anti-EV-A71 activity control. The infected RD cell monolayers incubated with cell culture medium supplemented with DMSO (infDMSO) served as negative anti-EV-A71 activity control. Uninfected RD cells incubated with cell culture medium alone (DMEM) and DMSO served as maximum % cell survival and vehicle control, respectively. The anti-EV-A71 activity is the ability to rescue the infected cells by determining % cell survival compared to the maximum % cell survival control. By the viral inoculum at M.O.I. of 1, the negative anti-EV-A71 activity control (infDMSO) showed 61.77 ± 8.44% cell survival compared to the uninfected cells, with maximum % cell survival control (Figure 2). Among positive anti-EV-A71 activity controls, the D-H7 (T-1106) exerted 71.18 ± 6.13% cell survival which was higher than those of D-A7 (rupintrivir), i.e., 67.36 ± 4.7%. Therefore, the cut-off point was set to 71.18% relative to those of D-H7 (T-1106). Because the detailed mechanism of the T-1106 on EV-A71 replication has not been elucidated, it was included in the subsequent experiment as a hit compound. The EV-A71 infected-RD cells treated with the compound D-D7 had 92.99 ± 5.94% cell survival which was higher than those of D-H2, D-H7, D-H8, E-C3, E-C4, and E-E5 ranging from 70.61 ± 1.42% to 77.77 ± 3.36%. It was suggested that the seven hit compounds might potentially have anti-EV-A71 activity as they can rescue the infected cells. They were investigated further for the inhibitory effects on EV-A71 replication. Characteristics of those hit compounds are described in Table 1.

### 2.3. Dissecting Mechanism of Inhibitory Effect of the Hit Compounds on EV-A71 Replication

**Figure 3:**
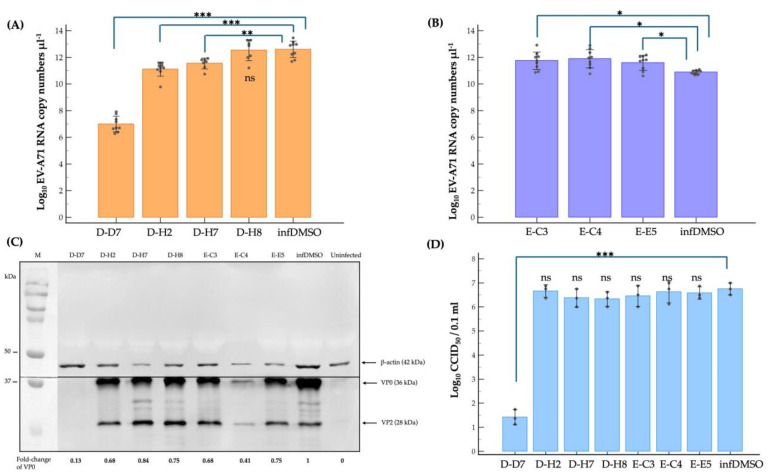
The hit compounds were verified for anti-EV-A71 activity by virus reduction assays for viral RNA copy numbers (**A**,**B**), viral protein synthesis (**C**), and mature particle production (**D**) using qRT-PCR, Western blot analysis, and CCID_50_ assay, respectively. The infected cells incubated with a cell culture medium supplemented with DMSO (infDMSO) were used as negative inhibition control and for comparison. The uninfected cells served as negative EV-A71 infection control (Uninfected). The log_10_ EV-A71 RNA copy numbers μL^−1^ and log_10_ CCID_50_/0.1 mL values of (**A**,**B**,**D**) are the means of three independent experiments. Error bars represent standard deviations. Each dark grey circle represents the individual data points. The VP0 fold-change values of (**C**) are representative of two independent experiments. Lane M is a protein marker. β-actin bands are used for protein normalization and internal loading control. Statistical analysis is the one-way analysis of variance (one-way ANOVA) with Tukey–Kramer pair-wise comparison and independent samples *t*-test as necessary. *, **, and *** represent a statistically significance difference at *p*-value < 0.05, *p*-value < 0.001, and *p*-value < 0.0001, respectively. ns indicates no significance.

The inhibitory effect of the D-D7, D-H2, D-H7, D-H8, E-C3, E-C4, and E-E5 compounds on the EV-A71 replication cycle was investigated in the infected-RD cells. The infected cells incubated with a cell culture medium supplemented with DMSO (infDMSO) were used as negative inhibition control and for comparison. The level of genome replication was quantified in the intracellular EV-A71 RNA by reverse-transcription quantitative real-time PCR (qRT-PCR) using a standard curve constructed from known amounts of recombinant plasmids inserted with EV-A71 VP1 coding sequence. Levels of viral protein synthesis were measured by measuring the EV-A71 unprocessed VP2-VP4 precursor protein, namely VP0, using Western blot analysis and densitometry. The calculated VP0 protein levels were compared with the infDMSO control and expressed as fold-change. A median cell culture infectious dose (CCID_50_) assay was used to titrate infectious mature EV-A71 particles [35]. Among seven compounds, only three compounds, including D-D7, D-H2, and D-H7, exhibited significant suppression of replication of the EV-A71 viral genome at approximately 5-log, 1-log, and 1-log reduction of copy numbers of intracellular viral RNA level, respectively (Figure 3A). The E-C3, E-C4, and E-E5 compounds significantly increased approximately 1-log increment of EV-A71 RNA copy numbers (Figure 3B). The EV-A71 protein synthesis was reduced significantly by the D-D7 and E-C4 compounds but marginally by the remaining five compounds (Figure 3C). Only the D-D7 compound could suppress the production of infectious EV-A71 particles at approximately a 5-log reduction of CCID_50_ (Figure 3D). The results suggested that some of the tested compounds could interfere with the replication cycle of EV-A71. Compound D-D7 (2-pyridone-containing peptidomimetics) exhibited the highest anti-EV-A71 activity.

## 3. Discussion

EV-A71 infection poses a major global public health challenge regarding fatal neurocomplications, high disease burden, and massive economic loss. Therefore, effective antiviral drugs against EV-A71 are needed. Unfortunately, despite striking advances in anti-EV-A71 drug discovery in the past 20 years, effective antiviral drugs against EV-A71 have not been found in clinical practice [12,13]. The traditional drug development approach commonly takes approximately 10 years from pre-clinical and clinical trial phases to license the approved drug for market availability [15]. Nevertheless, only 5% of drug candidates in the clinical trial phase are finally approved [15]. Hence, traditional drug development is time- and resource-consuming. To overcome these drawbacks, drug repurposing has become an alternative approach for drug discovery to treat viral infections. Drug repurposing of approved or investigational drugs is a strategy for identifying an alternative target or indication beyond the original scope [14,15]. By using drugs that are known for their safety profile, the phase I clinical trial could be skipped leading to a reduced attribution rate to be an approved antiviral drug. Hence, drug repurposing could significantly accelerate antiviral development for emergency use. Identification of repurposed drug candidates for the novel indication or target could be performed by in silico or in vitro screening [14,15]. For in silico screening, the method relies on computational analysis of the interaction between target and drug molecule. Hence, the data on the structure of the interacting molecules must be known or predictable. For in vitro screening, candidate compounds exhibiting potent antiviral activity can be high-throughput screened and validated mainly by a cell culture-based model. Hence, this study aimed to screen antiviral agents included in the Pandemic Response Box for repurposing to anti-EV-A71 activity and investigate the inhibitory effects of the compounds on viral replication by in vitro screening using a cell culture-based model.

The repurposed antiviral agents are categorized based on their inhibitory mechanism into two groups, including direct-acting repurposed antiviral (DARA) and host-targeting repurposed antiviral (HTRA) [15]. The virus- and host factors are essential for viral replication and have been targeted for developing potential anti-EV-A71 compounds in the investigational stages. The viral replication cycle is a multistep process composed of viral entry, genome uncoating, genome translation and replication, particle assembly (morphogenesis), and egression. After the EV-A71 gets into the host cell, its genome is directly translated and processed into viral proteins by virally encoded proteases, which subsequently replicate and translate the viral genome further, take control of cellular metabolism and biosynthesis processes, and subvert host immune responses that favor establishing productive viral infection.

Since viruses are obligate intracellular parasites, their replication depends solely on cellular factors and happens exclusively within the living host cells. The infecting virus must take control of cellular activities to favor its benefit using virally encoded proteins or complex structures of the genome. Therefore, antiviral drugs target the viral proteins or factors, namely DARA, which could inhibit virus replication. DARAs activity relies on structure similarity or identity of viral proteins shared among the related viruses, particularly RNA-dependent RNA polymerase (RdRp), proteases, helicase, ion conductance proteins, and capsid [12,13]. The DARAs of EV-A71 have been reported, including inhibitors of capsid, 3A, 2Cpro, 3Cpro, 3Dpol, and internal ribosomal entry site (IRES) [12,13]. DARAs that have inhibitory activity against viral enzymes could interfere with the respective steps in the replication cycle. Capsid inhibitors prevent conformational changes of the capsid, leading to inhibition of genome uncoating. IRES inhibitors block IRES-transacting factors (ITAFs) or canonical translational factors binding, resulting in suppression of viral genome translation.

Cellular proteins or pathways that are required and indispensable for the virus replication cycle are targets of HTRAs. Advantages of HTRAs include broad-spectrum antiviral activity and a high genetic barrier to drug resistance [15]. However, most antiviral drug discoveries have been focused on viral targets, and the most approved antiviral drugs are DARAs [15]. Importantly, the cytotoxicity offers a potential issue with host-targeting antivirals. For instance, chloroquine, an antimalarial drug, could inhibit EV-A71 replication in cell culture [15]. Even though the mechanism has not been exactly elucidated, the lysosomotropic property of chloroquine, which could inhibit lysosome functions, might be attributable to the uptake impairment of the virus. As a cellular pathway exploited by pan-enterovirus, the PI4KB-PI4P-OSBP pathway, which is crucial for the biogenesis of the viral replication organelles, has been reported to be a target of EV-A71 HTRA [15].

In this study, 153 antiviral agents at 1 μM, as recommended concentration by the MMV, were initially screened for cytotoxicity to RD cells and ability to rescue EV-A71-infected cells. There were seven hit compounds including 2-pyridone-containing peptidomimetics (D-D7), Selinexor (D-H2), T-1106 (D-H7), Clemizole (D-H8), Danirixin (E-C3), Pocapavir (E-C4), and Plerixafor (E-E5) had both negligible cytotoxicity towards RD cells and ability to rescue EV-A71-infected cells. It was found that some of the hit compounds could reduce EV-A71 genome replication and protein synthesis. Three hit compounds, including 2-pyridone-containing peptidomimetic, Selinexor, and T-1106, affect the genome replication of EV-A71. The original mode of action of 2-pyridone-containing peptidomimetics is a polyprotein processing inhibitor of human rhinovirus (HRV) 3C protease [26]. The crystal structure analysis shows the formation of an irreversible covalent bond between the inhibitor and the active site cysteine residue of HRV 3C, namely Cys147 [36]. This Cys147 residue is conserved among 3C proteins of picornaviruses, including EV-A71 and HRV [37]. Moreover, the similarity of the architecture of substrate binding cleft and mechanisms for substrate recognition between 3C proteases of HRV and EV-A71 has been revealed by superimposing their protein crystal structures [37]. Hence, the 2-pyridone-containing peptidomimetics can potentially bind the active site of EV-A71 3C protease. Therefore, the anti-EV-A71 activity of the 2-pyridone-containing peptidomimetic is likely an EV-A71 3C protease inhibitor. 3C protease is essential for the replication cycle of enteroviruses by cleaving the nascent polyprotein to generate functional viral proteins [37]. Hence, inhibition of EV-A71 3C protease results in a deficiency of functional viral proteins to translate and replicate the EV-A71 genome, leading to a drastic reduction of intracellular viral RNA, viral protein synthesis, and new virions. Because EV-A71 3C protease can impair innate immunity [38], the inhibitor seems to restore antiviral defenses to the viruses in the infected cells. This study has shed light on the 2-pyridone-containing peptidomimetics as a potent anti-EV-A71 drug. Selinexor, in combination with dexamethasone, has been an FDA-approved inhibitor of nuclear export protein exportin-1 (XPO1) to treat multiple myeloma [27]. Selinexor has been reported to inhibit the nuclear export of SARS-CoV-2 proteins, namely ORF3b, ORF9b, and nucleocapsid and ACE-2 host receptors, and suppress the virus replication [39]. The genome of EV-A71 requires ITAFs to interact with IRES and drive cap-independent translation. Selinexor might interfere with the nuclear export of some ITAFs interacting with EV-A71 IRES, resulting in a slight reduction in viral protein synthesis and intracellular viral RNA. Hence, the production of new virions was not affected by the Selinexor treatment. Moreover, the related paper demonstrated that Selinexor downregulated the pro-inflammatory cytokines commonly associated with the cytokine storm observed in COVID-19 patients [39]. It has been postulated that immunopathology might be the pathogenesis mechanism of neurocomplications associated with EV-A71 infection [40]. Therefore, the modulation of inflammation in EV-A71 infected cells by Selinexor should be investigated. T-1106, a nucleoside analog, is a nucleoside version of Favipiravir lacking fluorine [41]. Metabolism of the T-1106 to the triphosphate yields a drug with efficacy superior to favipiravir. The mode of function of general nucleoside analogs is to induce lethal mutagenesis or chain termination [20]. Unlike those nucleoside analogs, the T-1106, once incorporated into the EV-A71 genome by the RdRp enzyme, causes the enzyme to pause and backtrack, promoting template switching and the formation of defective viral genomes [20]. However, the therapeutic effect of T-1106 that could inhibit EV-A71 replication in RD cells required very high concentrations between 200 μM and 600 μM [20]. Our study used 1 μM T-1106. Hence, it could slightly suppress EV-A71 RNA replication and protein synthesis, which insufficiently affects virion production. Pocapavir is an enterovirus-specific capsid inhibitor [33]. It has been used as an investigational drug for the treatment of severe neonatal enteroviral sepsis due to coxsackievirus B3 [42]. It also has been treated for chronic echovirus 11 meningoencephalitis in an immunocompromised adult patient [43]. Moreover, Pocapavir has been reported for clearance of vaccine-derived type 3 poliovirus infection in an infant with underlying X-linked agammaglobulinemia [44]. The binding site of Pocapavir is within the pocket located in the structural protein VP1, namely canyon [33]. The Pocapavir binding results in an increase in the stability of the viral capsid, thus preventing conformational changes necessary for receptor interaction and uncoating. Hence, it acts in the early stages of the enterovirus replication cycle. Related papers determined that EV-A71 genome uncoating was initiated at 20 min post-infection in RD cells and completed within 2 h [45]. The increase in intracellular viral RNA was found at 2 h post-infection followed by virus secretion at 8 h post-infection [45]. In this study, the EV-A71-infected RD cells were added with Pocapavir at 1 h post-infection, in which the viruses might initiate the replication cycle at receptor binding and genome uncoating stages already. Hence, the binding sites of Pocapavir are already masked by receptor engagement. Consequently, Pocapavir did not exhibit an inhibitory effect on virion production in our model. Even though the VP0 (precursor of VP2 and VP4) does not contain the inhibitor-binding site, the protein level was significantly reduced by Pocapavir treatment. It could be suggested that the biogenesis of intracellular EV-A71 capsid protein might be interfered with by Pocapavir. Clemizole, Danirixin, and Plerixafor did not exhibit anti-EV-A71 activity. They showed only a slight interference effect on the VP0 protein level. It could be speculated that those three hit compounds might interfere with host factors involving the biogenesis of intracellular EV-A71 capsid protein. Interestingly, EV-A71 RNA levels were increased in the infected RD cells treated with Danirixin, Pocapavir, and Plerixafor. Related articles show that intracellular EV-A71 VP1 induces autophagy to regulate viral replication [46]. We hypothesized that the interaction between intracellular VP1 and Pocapavir might enhance VP1-induced autophagy activity. Danirixin and Plerixafor are antagonists of CXCR2 and CXCR4 chemokine receptors, respectively. CXCR2 and CXCR4 are G protein-coupled receptors (GPCR) engaging with several downstream signaling pathways that facilitate various tissue-dependent signals, including cell migration, adhesion, proliferation, survival, and differentiation [47,48]. It has been reported that CXCR2 and CXCR4 can induce apoptosis [48,49]. Apoptosis regulation is important in EV-A71 replication [50]. Hence, in this study, we speculated that apoptosis could be suppressed upon Danirixin and Plerixafor binding with their cognate chemokine receptor antagonists, resulting in enhanced EV-A71 RNA replication. Besides the effects of the hit compounds, compensatory mechanisms mediated by the EV-A71 might involve the abovementioned phenomena. Those inconclusive mechanisms warrant further experimental validations.

This study evaluated the therapeutic effect of the 2-pyridone-containing peptidomimetics, Selinexor, T-1106, Clemizole, Danirixin, Pocapavir, and Plerixafor but not prophylactic effect. Moreover, in this study, hit compounds’ median effective concentration (EC50), 50% cytotoxic concentration (CC50), and selectivity index (SI) were not determined. It was envisaged that those parameters be defined to obtain hit compounds’ potency, safety, and efficacy information. The remaining 201 antibacterial and 46 antifungal compounds in the Pandemic Response Box should be screened for anti-EV-A71 activity to gain more repurposed drugs for further investigation and development. The anti-enteroviral activity of the compounds was not tested against other EV-A71 genotypes. The abovementioned limitations warrant further investigations. The future direction of the gained information is to study the pharmacokinetics and pharmacodynamics of the compounds in animal models. Modifications of the chemical structure of the compounds and their synergistic effects could improve their antiviral effect. These actions can facilitate the successful licensing of anti-EV-A71 drugs for clinical practice.

## 4. Materials and Methods

### 4.1. Cell Culture and Virus

Rhabdomyosarcoma (RD) cells (JCRB9072) were purchased from the Japanese Collection of Research Bioresources Cell Bank and grown in Dulbecco’s modified Eagle’s medium (DMEM, Gibco, New York, NY, USA) supplemented with 1× GlutaMAX (Gibco, New York, NY, USA), 10% heat-inactivated fetal bovine serum (HyClone, Waltham, MA, USA), L-glutamine, and penicillin/streptomycin antibiotics (Gibco, New York, NY, USA), namely completed DMEM. The cells were incubated at 37 °C in the humidified air containing 5% CO_2_.

Enterovirus A71 genotype B5 (Thai patient isolate) [51] stocks were produced on RD cells as described previously [35] and aliquoted to store at −80 °C. The viral titers were determined by median cell culture infectious dose (CCID_50_) assay and calculated by Kärber formulation expressed as described previously [35].

### 4.2. Compound Preparation of the Pandemic Response Box

The 153 antiviral compound stocks (purity of >90%) included in the Pandemic Response Box were dissolved in 100% DMSO (*v*/*v*) at 10 mM in 96-well plates [21]. The compound stocks were 1:10,000 diluted in the completed DMEM to prepare 1 μM working compounds. The working compounds were aliquoted and kept at −80 °C until further use. The 0.01% DMSO in the completed DMEM was used as a vehicle or negative control.

### 4.3. Sulforhodamine B (SRB) Assay

Cell viability of RD cells treated with compounds was assessed for cytotoxicity and inhibitory activity on EV-A71-induced cytopathic effect (CPE) by SRB assay [52]. Briefly, RD cell suspension was added in wells of a 96-well plate (10^4^ cells per well). The RD cell monolayers that reached approximately 70% to 80% confluence were incubated with 1 μM working compounds and kept for 24 h to test the cytotoxicity of the compounds. Cell monolayers incubated with the completed DMEM and the 0.01% DMSO diluted in the completed DMEM served as maximum % cell survival and vehicle control, respectively. To test inhibitory activity on EV-A71-induced CPE, RD cell monolayers were adsorbed with 1 M.O.I. EV-A71 was prepared in serum-free DMEM for 1 h, then washed and replenished with fresh completed medium containing 1 μM working compounds. The treated infected cells were incubated for 24 h. The infected cells incubated with the 0.01% DMSO diluted in the completed DMEM (infDMSO) were negative anti-EV-A71 activity control. The uninfected RD cells incubated with the completed DMEM and the 0.01% DMSO diluted in the completed DMEM served as maximum % cell survival and vehicle control, respectively. At the end-point, 25 μL of iced-cold 50% (*w*/*v*) trichloroacetic acid (Sigma-Aldrich, St. Louis, MO, USA) was directly added to the wells for fixing the cultured cells. The plates were kept at 37 °C for 30 min, and the fixed cell monolayers were then washed with distilled water and air-dried at an ambient temperature. The intracellular protein contents of the survived cells were stained by adding the wells with 50 μL of 0.04% (*w*/*v*) SRB solution (Sigma-Aldrich, St. Louis, MO, USA) and kept at an ambient temperature for 1 h. After that, the excess dyes were removed by rinsing the fixed cell monolayers with 1% (*v*/*v*) acetic acid and air-dried. The intracellular protein-bound SRB dyes were dissolved in 100 μL of 10 mM Tris base solution pH 10.5. The color intensity of the eluates was determined by measuring absorbance at optical density at 510 nm (OD_510nm_) using a FlashScan™ microplate reader (Thermo Fisher Scientific, Vantaa, Finland). The percentages of cell survival were calculated using the formula: % cell survival = (OD_510nm_ of test or irrelevant control ÷ OD_510nm_ of maximum % cell survival control) × 100.

### 4.4. Virus Reduction Assays

The hit compounds were verified the anti-EV-A71 activity by virus reduction assays, including reverse-transcription quantitative real-time PCR (qRT-PCR), Western blot analysis, and CCID_50_ assay for quantifying viral RNA copy numbers, viral protein synthesis, and mature particle production, respectively. The RD cell monolayers were infected with 1 M.O.I. EV-A71, treated with 1 μM compounds, and the negative anti-EV-A71 activity control (infDMSO) were prepared as described in Section 4.3. At 24 h post-treatment, cell culture supernatants were collected to perform the CCID_50_ assay [35,53]. The cell monolayers were washed once with sterile phosphate-buffered saline and added with 500 µL Trizol reagent (Invitrogen, Waltham, MA, USA) to extract protein and total RNA according to the manufacturer’s instructions. The concentration of the prepared protein lysates was measured using a Bradford protein assay. The concentration and purity of total RNA were measured using a spectrophotometer.

The protein lysates were subjected to Western blot analysis to measure EV-A71 capsid protein VP0 (VP2-VP4 precursor) levels and host β-actin protein as described previously [53]. One microgram of protein lysates was separated by sodium dodecyl sulfate-polyacrylamide gel electrophoresis (SDS-PAGE) using denaturing discontinuous polyacrylamide gel consisting of two layers of 12% resolving gel and 4% stacking gel (Bio-Rad Laboratories, Hercules, CA, USA). The SDS-PAGE-separated proteins were transferred to nitrocellulose membranes (Bio-Rad Laboratories). The empty areas on the blotted membranes were blocked with 5% (*v*/*v*) fetal bovine serum (FBS) diluted in phosphate-buffered saline (PBS) for 1 h. Then, the membranes were washed with PBS containing 0.05% (*v*/*v*) Tween-20, namely PBS-T. After that, the membranes were separately incubated with 1:3000 mouse anti-β-actin antibodies (cat. BF0198, Affinity Biosciences, Cleveland, OH, USA) and 1:3000 mouse anti-enterovirus 71 antibodies (cat. 4175-0127, Bio-Rad Laboratories) for 1 h. After washing, the membranes were incubated with 1:3000 goat anti-mouse IgG (H + L) conjugated with horseradish peroxidase (KPL, SeraCare, Milford, MA, USA) at room temperature for 1 h. All antibodies were diluted in 0.2% FBS (*v*/*v*) in PBS-T. The membrane was incubated with Clarity Max Western ECL substrate (Bio-Rad Laboratories) at room temperature for 10 min and imaged by ChemiDocTM XRS+ (Bio-Rad Laboratories) to visualize the protein bands. The protein levels of EV-A71 VP0 and β-actin were determined by measuring the protein band intensities using Image LabTM Software Version 6.1 (Bio-Rad Laboratories). Then, the protein levels of EV-A71 VP0 were normalized with those of β-actin in each sample. The fold changes of normalized VP0 were relative to negative anti-EV-A71 activity control [53].

Following the instruction protocol, one microgram of total RNA of each sample was treated with DNase I (Thermo Fisher Scientific, Waltham, MA, USA). The RNA samples were diluted to 200 ng/μL, and the same amounts of RNA were used in the qRT-PCR. Recombinant pQE31 plasmids inserted with EV-A71 VP1 coding sequence, namely pQE31::VP1 constructed previously [51], were 10-fold serially diluted to yield 10 to 10^6^ copies of the VP1 for creating a standard curve. The qRT-PCR reactions of the RNA samples and pQE31::VP1 were prepared in one-step SYBR Green-based qRT-PCR reagents (Agilent, Santa Clara, CA, USA) using EV-A71 VP1 specific primers (EV-F2760 forward primer; 5′-ATGGKTATGYWAAYTGGGACAT-3′, and EV-R3206 reverse primer; 5′-CCTGACRTGYTTMATCCTCAT-3′) following the protocol described previously [53]. The copy numbers of viral RNA were calculated from cycle threshold (Ct) values using the standard curve. The lower limit of detection was estimated as 10^2^ copies of the VP1.

### 4.5. Statistical Analysis

MedCalc^®^ Statistical Software, Version 22.014, was used for all analyses (MedCalc Software Ltd., Ostend, Belgium). The VP0 fold-change values were representative of two independent experiments. All SRB assay and CCID_50_ data were derived from three independent experiments. The qRT-PCR data were derived from technical triplicates of each sample conducted by three independent experiments. They were expressed in mean ± standard deviations (SD). The mean ± SD values of qRT-PCR and CCID_50_ data of the compound-treated groups were compared with the infDMSO groups using one-way analysis of variance (one-way ANOVA) with Tukey–Kramer pair-wise comparison and independent samples *t*-test as necessary. A *p*-value less than 0.05 indicated a statistically significant difference.

## Figures and Tables

**Table 1:** Chemical structures, alternative names, and mode of action of the selected hit compounds.

**Compound** **(MMV ID) [21]**	**ChEMBL ID**	**Trivial Name**	**Formula**	**Structure [22,23,24,25]**	**Mode of Action**
D-D7 (MMV1782112)	CHEMBL 141816	2-pyridone-containing peptidomimetics	C_25_H_31_N_5_O_7_	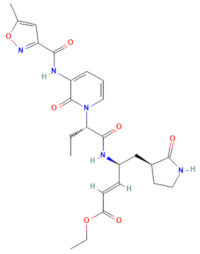	Polyprotein processing inhibitor of human rhinovirus 3C protease [26]
D-H2 (MMV1593517)	CHEMBL 3545185	Selinexor	C_17_H_11_F_6_N_7_O	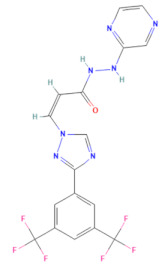	Nuclear export protein inhibitor of CRM1 or XPO1 [27]
D-H7 (MMV1782104)	CHEMBL 261459	T-1106	C_10_H_13_N_3_O_6_	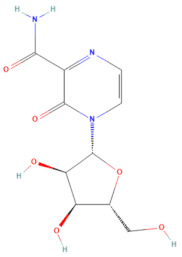	RNA polymerase inhibitor of EV-A71 [20]
D-H8 (MMV002015)	CHEMBL 1407943	Clemizole	C_19_H_20_ClN_3_	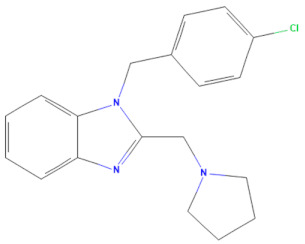	NS4B protease inhibitor of HCV [28]
**Compound** **(MMV ID) [21]**	**ChEMBL ID**	**Trivial Name**	**Formula**	**Structure [29,30,31]**	**Mode of Action**
E-C3 (MMV1581034)	CHEMBL 3039531	Danirixin	C_19_H_21_ClFN_3_O_4_S	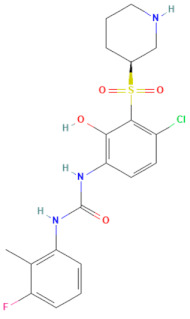	Reversible antagonist of CXCR2 chemokine receptor [32]
E-C4 (MMV1580485)	CHEMBL 1235858	Pocapavir	C_21_H_17_Cl_3_O_3_	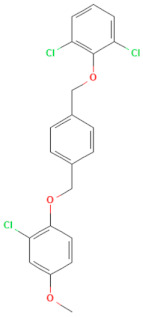	Viral capsid inhibitor of enteroviruses [33]
E-E5 (MMV1580502)	CHEMBL 18442	Plerixafor	C_28_H_54_N_8_	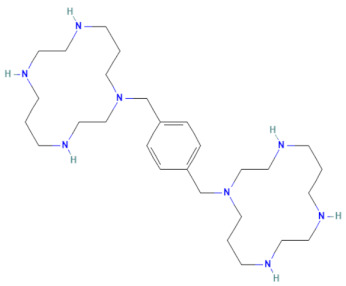	CXCR4 chemokine receptor antagonists [34]

## Data Availability

Data are contained within the article.
